# Gene co‐expression patterns in Atlantic salmon adipose tissue provide a molecular link among seasonal changes, energy balance and age at maturity

**DOI:** 10.1111/mec.17313

**Published:** 2024-03-01

**Authors:** Ehsan Pashay Ahi, Jukka‐Pekka Verta, Johanna Kurko, Annukka Ruokolainen, Pooja Singh, Paul Vincent Debes, Jaakko Erkinaro, Craig R. Primmer

**Affiliations:** ^1^ Organismal and Evolutionary Biology Research Programme, Faculty of Biological and Environmental Sciences University of Helsinki Helsinki Finland; ^2^ Department of Aquatic Ecology, Institute of Ecology and Evolution University of Bern Bern Switzerland; ^3^ Center for Ecology, Evolution & Biogeochemistry Swiss Federal Institute of Aquatic Science and Technology (EAWAG) Kastanienbaum Switzerland; ^4^ Department of Aquaculture and Fish Biology Hólar University Sauoarkrokur Iceland; ^5^ Natural Resources Institute Finland (Luke) Oulu Finland; ^6^ Institute of Biotechnology, Helsinki Institute of Life Science (HiLIFE) University of Helsinki Helsinki Finland

**Keywords:** adipose tissue, Atlantic salmon, ecology, gene expression, Hippo pathway, seasonality, sexual maturation, *vgll3*

## Abstract

Sexual maturation in many fishes requires a major physiological change that involves a rapid transition between energy storage and usage. In Atlantic salmon, this transition for the initiation of maturation is tightly controlled by seasonality and requires a high‐energy status. Lipid metabolism is at the heart of this transition since lipids are the main energy storing molecules. The balance between lipogenesis (lipid accumulation) and lipolysis (lipid use) determines energy status transitions. A genomic region containing a transcription co‐factor of the Hippo pathway, *vgll3*, is the main determinant of maturation timing in Atlantic salmon. Interestingly, *vgll3* acts as an inhibitor of adipogenesis in mice and its genotypes are potentially associated with seasonal heterochrony in lipid storage and usage in juvenile Atlantic salmon. Here, we explored changes in expression of more than 300 genes directly involved in the processes of adipogenesis, lipogenesis and lipolysis, as well as the Hippo pathway in the adipose tissue of immature and mature Atlantic salmon with distinct *vgll3* genotypes. We found molecular evidence consistent with a scenario in which immature males with different *vgll3* genotypes exhibit contrasting seasonal dynamics in their lipid profiles. We also identified components of the Hippo signalling pathway as potential major drivers of *vgll3* genotype‐specific differences in adipose tissue gene expression. This study demonstrates the importance of adipose gene expression patterns for directly linking environmental changes with energy balance and age at maturity through genetic factors bridging lipid metabolism, seasonality and sexual maturation.

## 1 INTRODUCTION

Ecological factors play fundamental roles in the maintenance of variation in life‐history genes (Jops & O'Dwyer, 2023), particularly those linked with ‘pace‐of‐life’ traits (Arnqvist & Rowe, 2023). The timing of sexual maturation (age at maturity) is a crucial life‐history trait influenced by environmental cues as well as genetic and physiological mechanisms regulating the onset of puberty (Taranger et al., 2010). Age at maturity also subsequently affects other crucial fitness traits such as survival and reproductive success (Mobley et al., 2021). Elucidating the molecular basis of variation in age at maturity, especially in relation to environmental cues, is thus a pivotal step towards understanding the maintenance of the diversity in life‐history traits and its ecological significance.

Sexual maturation in Atlantic salmon is tightly linked with seasonal environmental changes. For example, temperature changes can affect metabolism and body condition, via lipid allocation, by impacting food availability, and thereby growth rate (House, Debes, Kurko, et al., 2023). Lipid allocation has been found to be especially important for age at maturity in Atlantic salmon due to their need to reach a certain threshold of lipid reserves in order to initiate maturation (Rowe et al., 2011). This allocation process can be strongly affected by variation in replenishing reserves across seasonal changes, including periods of low temperatures and food availability (Mogensen & Post, 2012). In Atlantic salmon, a single locus including the gene vestigial‐like family member 3 (*vgll3*) is the major genetic determinant of maturation timing (explaining over 39% of the variation in the age at maturity) in a sex‐specific manner (Barson et al., 2015; Czorlich et al., 2018; Miettinen et al., 2023). The effects of *vgll3* on age at maturity can be observed in males as early as 1 year of age in controlled conditions (Debes et al., 2021; Sinclair‐Waters et al., 2021; Verta et al., 2020). Interestingly, the human orthologue (*VGLL3*) has also been shown to be associated with age at maturity (Cousminer et al., 2016; Perry et al., 2014). In addition to being linked with maturation age, *vgll3* has also been shown to function as an inhibitor of adipogenesis in mice (Halperin et al., 2013). In salmon, energy storage in individuals with the distinct *vgll3* genotypes varies across seasons thereby providing an interesting link between energy expenditure and environmental change (House, Debes, Kurko, et al., 2023).

Recent studies of 1‐year‐old Atlantic salmon identified strong association between *vgll3* early (E) and late (L) maturation alleles and the expression of key reproductive axis genes (Ahi et al., 2022, 2024). Gene co‐expression network (GCN) analysis predicted that the *vgll3* genotype effects on reproductive axis genes are likely to be mediated by differential activation of the Hippo signalling pathway (Ahi et al., 2023). The Hippo pathway is emerging as a key molecular signal not only in controlling sexual maturation in vertebrates (Kjærner‐Semb et al., 2018; Kurko et al., 2020; Sen Sharma et al., 2019) but also in balancing adipocyte proliferation versus differentiation. The activity of Yap1, a major transcription co‐factor of the Hippo pathway, has been found to be indispensable during adipogenesis (Ardestani et al., 2018). Strikingly, Hippo pathway appears to be an essential molecular cue for responding to environmental effects, such as changes in diet fat and temperature, at transcriptional levels (Luo et al., 2020; Shu et al., 2019). Vgll3 acts as a major activating transcription co‐factor of the Hippo pathway and competes with the other major transcription co‐factor of the pathway, Yap1, which mainly acts as an inhibitor of the Hippo pathway (Hori et al., 2020; Kurko et al., 2020). Together, this suggests that Atlantic salmon, with distinct *vgll3* alleles tightly linked to maturation and adipogenesis processes, are an excellent natural model system to explore molecular mechanisms directly linking sexual maturation, energy acquisition and environmental changes.

In this study, we used a custom‐made Nanostring gene expression panel to investigate the expression patterns of 337 genes including genes encoding adipogenesis related factors, Hippo pathway components and their associated interacting partners/signals in the adipose tissue of mature and immature male Atlantic salmon with *early* (E) or *late* (L) maturing *vgll3* genotypes. Comparisons between maturity status and *vgll3* genotype enabled application of pathway‐ and regulatory network‐based approaches for better understanding underlying molecular processes.

## 2 MATERIALS AND METHODS

### 2.1 Fish material and tissue sampling

Individuals used in the study were from the same population (Oulujoki) and cohort used in Verta et al. (2020). This provided access to individually passive integrated transponder‐tagged individuals with known *vgll3* genotypes (see Verta et al., 2020 for more details of crossing and rearing). For this study, male individuals were sampled at the ages of 1.5–2 years post‐fertilisation (Debes et al., 2020). Following euthanisation with an overdose of MS222, various tissues, including visceral adipose tissue, of individual males were sampled at three time points, each reflecting a different maturation development stage:

#### 2.1.1 Immature 1

Individuals were sampled in late spring (5–21 May), average mass 17.5 g (range 10.2–27.4 g), average length 12.1 cm (range 9.1–13.6 cm), and showed no indications of gonad development (gonadosomatic index; GSI = 0).

#### 2.1.2 Immature 2

Individuals were sampled in the summer (4–17 July), average mass 33.7 g (range 21.1–76.6 g), average length 17.3 cm (range 14–18.5 cm), and some individuals showed initial signs of the commencement of phenotypic maturation processes (GSI = 0.0–0.4).

#### 2.1.3 Mature

Individuals were sampled during the expected spawning period in the early autumn (1–15 October), average mass 82.7 g (range 60.3–120.6 g), average length 20.8 cm (range 16.4–22.7 cm), and all individuals had well‐developed gonads reflective of being mature (GSI >3).

These tissues were flash frozen in liquid nitrogen and stored at −80°C until processing.

### 2.2 RNA extraction

In total, RNA was extracted from 24 samples of male visceral adipose tissue using a NucleoSpin RNA kit (Macherey‐Nagel GmbH & Co. KG). The samples were transferred to tubes with 1.4‐mm ceramic beads (Omni International), Buffer RA1 and DDT (350 μL RA1 and 3.5 μL DDT 1 M) and homogenised for 2 min (6 × 20 s) at 30 Hz using Bead Ruptor Elite (Omni International). The RNA extraction steps were conducted according to the manufacturer's instructions. The kit also contained a built‐in DNase step to remove residual gDNA. At the end, RNA extracted from each sample was eluted in 50 μL of nuclease free water. RNA quantity was measured with NanoDrop ND‐1000 (Thermo Scientific, Wilmington, DE, USA) and quality was assessed with 2100 BioAnalyzer system (Agilent Technologies, Santa Clara, CA, USA), and the RNA integrity number was above 7 in all the samples. From each extraction, 100 ng of total RNA was used for hybridisation step in the Nanostring panel.

### 2.3 Nanostring nCounter mRNA expression panel

NanoString nCounter is a multiplex nucleic acid hybridisation technology that enables reliable and reproducible assessment of the RNA expression of up to several hundred genes in a single assay (Goytain & Ng, 2020). This technology has several advantages for ecological and evolutionary research as it requires very small amount of RNA input with lower quality than RNA‐seq, does not have an amplification step and it can detect very low RNA expression levels. The Nanostring panel of probes used in the current study was an extended panel from that used in Kurko et al. (2020) that was previously developed to investigate age at maturity related gene expression in Atlantic salmon (more than 100 genes added). These genes include an extensive list of Hippo pathway components and other known genes having direct cross‐talk with the Hippo pathway. The selection was conducted based on the literature and the IPA (Ingenuity Pathway Analysis) tool (Qiagen) and other freely available web tools and databases (Kurko et al., 2020). In addition, the panel contains probes for the age‐at‐maturity‐associated genes in Atlantic salmon; *vgll3a* on chromosome 25 and *six6a* on chromosome 9, and their corresponding paralogues *vgll3b* on chromosome 21 and *six6b* on chromosome 1. Further, probes for other functionally associated genes with important roles in metabolism (e.g. lipidogenesis), cell fate (adipogenesis) and sexual maturation (e.g. HPG axis), were also included. Most of the candidate genes possess one or more paralogues due to the recent Atlantic salmon genome duplication, and therefore, paralogues of each gene of interest were also included after identification through the SalmoBase (http://salmobase.org/), and NCBI RefSeq databases. Further details on the selection of the genes/paralogues and naming of them can be found in Kurko et al. (2020). Gene accession numbers, symbols, full names and functional categories are listed in File S1. The mRNA expression levels of the candidate genes were investigated using Nanostring nCounter Analysis technology (NanoString Technologies, Seattle, WA, USA). Probes for each gene paralogue, targeted at all known transcript variants, were designed using reference sequences in the NCBI RefSeq database. However, for some genes, it was not possible to design paralogue‐specific probes, as sequence similarity between paralogues was too high. The RNA samples were analysed using nCounter Custom CodeSet for probes and nCounter Master kit (NanoString Technologies). The RNA of each sample was denatured, hybridised with the probes overnight and in the following day post‐hybridisation purification and image scanning were conducted.

### 2.4 Data analysis

Among nine candidate reference genes in the panel, seven genes, including *ef1a* paralogues (*ef1aa*, *ef1ab* and *ef1ac*), *hprt1*, *prabc2* paralogues (*prabc2a* and *prabc2b*) and *rps20*, were selected for data normalisation since they showed a low coefficient of variation (CV) values across the samples. The excluded reference genes were *actb* and *gapdh* showing very high variation (CV% >100), and even though they are commonly used as reference genes in many studies, they seemed to be unsuitable for data normalisation in the adipose tissue of Atlantic salmon. Subsequently, the raw count data from the Nanostring nCounter mRNA expression was normalised by RNA content normalisation factor for each sample calculated from geometric mean count values of the selected seven reference genes. After normalisation, a quality control step was conducted on the data and all the samples passed the default limit based on the nSolver Analysis Software v4.0 (NanoString Technologies; www.nanostring.com/products/nSolver). Mean of the negative controls were subtracted while analysing the data using the software and positive control normalisation was performed using the geometric mean of all positive controls as recommended by the manufacturer. A normalised count value of 20 was set as a background signal threshold, and consequently, 121 genes had on average below background signal across the samples, which left 202 genes to be considered for further analyses. The differential expression analysis was performed using the log‐linear and negative binomial model (lm.nb function), as implemented in Nanostring's nSolver Advanced Analysis Module (nS/AAM). The maturation status and genotypes were selected as predictor covariates in the model as suggested by nS/AAM. Multiple hypothesis adjustment was performed using the Benjamini–Yekutieli method (Benjamini & Yekutieli, 2001) within the software and adjusted *p*‐values <.05 were considered significant (File S2). The log‐transformed expression values were also used for calculations of pairwise Pearson correlation coefficients (*r*) between the expression of each gene and GSI values across all the samples.

The Weighted Gene Coexpression Network Analysis (WGCNA version 1.68) R‐package (version 5.2.1) was implemented to identify GCNs (Langfelder & Horvath, 2008). Since our main interest was in the comparison of alternative *vgll3* genotypes, all samples from both maturation statuses and time‐points within each genotype were used as biological replicates, providing sufficient statistical power for WGCNA. To identify sample relationships, we conducted hierarchical clustering of samples based on gene expression. Coexpression networks were constructed with the following steps: (1) calculation of Pearson correlation coefficients to identify and measure gene coexpressions, (2) calculation of an adjacency matrix (with reference to a scale free topology) using the coefficients, (3) calculation of the topological overlap distance matrix using the adjacency matrix, (4) hierarchically clustering of genes (method = average) using the topological overlap distance, (5) identification of coexpressed modules of genes using the cutTreeDynamic function with a minimum module size of 10 genes (6) colour assignment for each module and representation module‐specific expression profile by the first principal component (module eigengene, ME) of each module, and (7) merging highly similar modules based on ME dissimilarity (distance threshold of 0.25) in order to find the final set of coexpressed gene modules. Next, a conditional coexpression analysis was set (as described by Singh et al., 2021); that is, coexpression networks were constructed for each *vgll3* genotype separately, to identify the preservation of EE modules in the LL network and vice versa. A soft‐power of 7 was used to construct adjacency matrix. Finally, the module preservation statistics were calculated using WGCNA to test how the density and connectivity of modules defined in the reference dataset (e.g. EE) were preserved in the query dataset (e.g. LL) (Langfelder et al., 2011). A permutation test was implemented to repeatedly permute genes in the query network in order to calculate Zscore. Individual Z scores from all permutations (200) were summarised as a Zsummary statistic.

Gene ontology (GO) enrichment analysis was conducted to identify overrepresented biological process in each GCN using Manteia (Tassy & Pourquié, 2014). The GO enrichment criteria were limited to false discovery rate (FDR) <0.05 (false discovery rate) with GO specificity level of 2 set as a cut‐off. To predict potential gene interactions and key genes with the highest number of interactions (interacting hubs), the identified differentially expressed genes in each comparison were converted to their conserved orthologues in human (which have the highest amount of validated/studied interactome data across vertebrates) and used as input for STRING version 12.0, one of the largest freely available knowledge‐based interactome database for vertebrates (Szklarczyk et al., 2023). The interaction predictions between genes were based on data from structural similarities, cellular co‐localisation, biochemical interactions and co‐regulation. The confidence level to predict each interaction/molecular connection was set to medium (the default).

## 3 RESULTS

### 3.1 Differences in gene expression between *vgll3* genotypes

**FIGURE 1:**
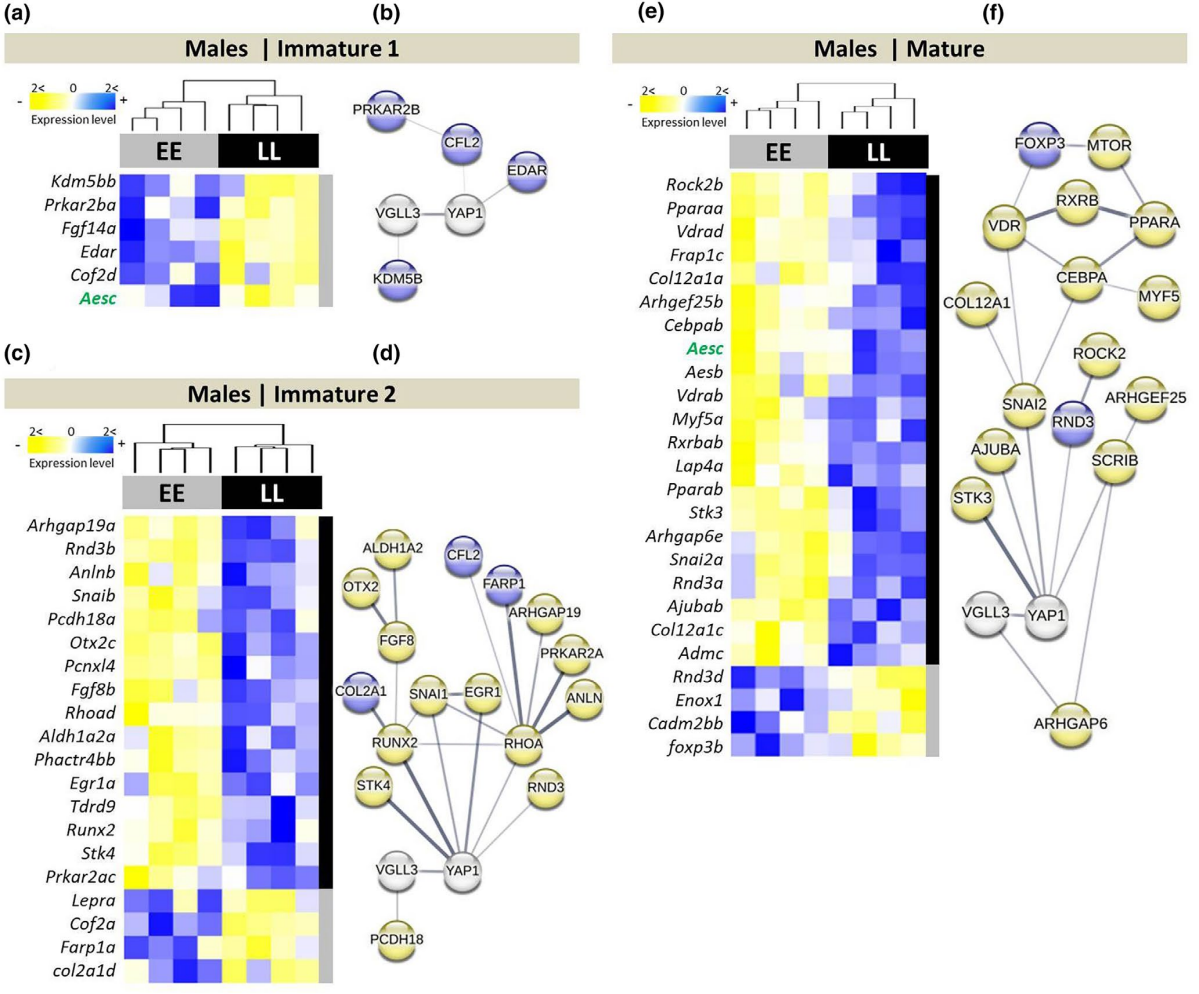
Differentially expressed genes between alternative *vgll3* genotypes and their predicted interactions in Atlantic salmon adipose tissue. Heatmaps represent differentially expressed genes between *vgll3* genotypes at three timepoints (a, c, e) and their respective predicted interactions using STRING v12 (http://string‐db.org/) (b, d, f). The thickness of the connecting lines between the genes indicates the probability of the interaction. Genes coloured in blue and yellow in the predicted network indicate higher and lower expression in vgll3*EE individuals, respectively.

We first assessed expression differences between *vgll3* genotypes in each developmental stage; two stages of *Immature 1* and *2* (late spring and summer) and *Mature* (early autumn) (Figure 1). We found six differentially expressed genes at *Immature 1*, and 20 genes at *Immature 2* time point, as well as 25 genes at *Mature* time point between the genotypes. At *Immature 1* (late spring), all of the six differentially expressed genes identified showed higher expression in vgll3*EE genotype individuals (Figure 1a). A query of molecular interactions revealed one gene, *kdm5bb*, showing direct interaction with *vgll3*, and two genes with *yap1* (*edar* and *cof2d/cfl2*) (specified with connecting lines between the genes in Figure 1b). At *Immature 2*, we found 16 genes with higher expression in vgll3*LL genotype individuals and four genes showing higher expression in vgll3*EE genotype individuals (Figure 1c). The predicted interactions revealed five genes (*rhoad*, *rnd3b*, *runx2*, *snaib* and *stk4*) with direct molecular interaction with *yap1* whereas one gene (*pcdh18c*) had direct interaction with *vgll3* (Figure 1d). Furthermore, three of the differentially expressed genes (*rhoad*, *runx2* and *snaib*) formed interacting hubs (they showed the highest number of interactions of all investigated genes) and *rhoad* displayed the highest number of connections indicating its potential key functional role in this interaction network (Figure 1d). Finally, at *Mature*, we found 21 genes with higher expression in vgll3*LL genotype individuals, and four genes showed higher expression in vgll3*EE genotype individuals (Figure 1e). The interaction query identified five genes (*ajubab*, *lap4a*/*scrib*, *rnd3a*, *snai2a* and *stk3*) with direct interactions with *yap1*, whereas one gene (*arhgap6a*) had a direct interaction with *vgll3* (Figure 1f). One of these differentially expressed genes, *snai2a*, formed an interacting hub, and also two of its interacting genes, *vdrad* and *cebpab*, appeared to make interacting hubs by connecting to three other genes (Figure 1f).

### 3.2 Maturation‐specific gene expression differences

**FIGURE 2:**
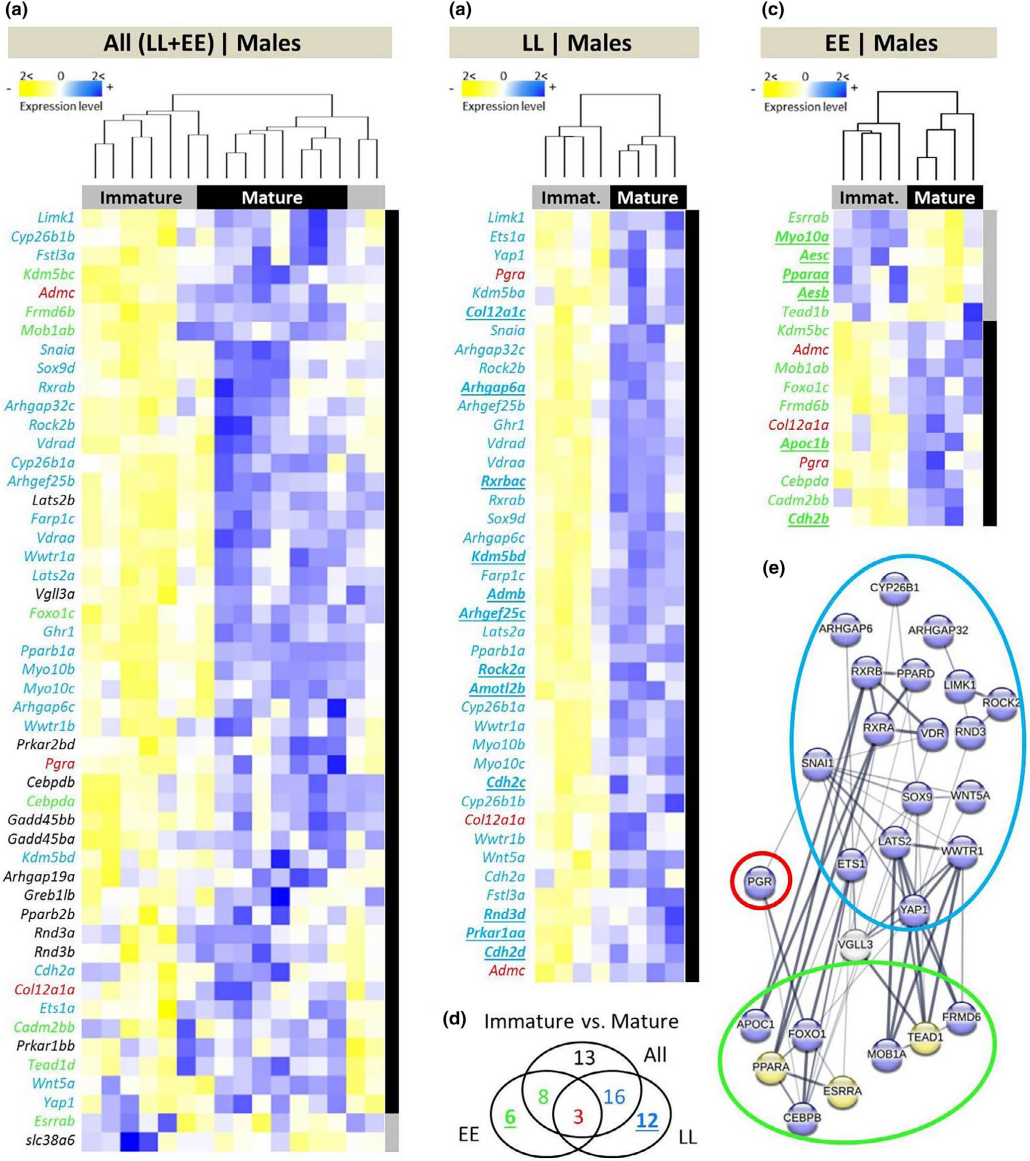
Differentially expressed genes between immature versus mature male Atlantic salmon and their predicted interactions in adipose tissue. Heatmaps representing differentially expressed genes between the immature (those individuals with GSI = 0) versus mature males across alternative *vgll3* homozygotes (a), and within vgll3*LL (b) and vgll3*EE genotype individuals (c). A Venn diagram showing the numbers of differentially expressed genes overlapping between the comparisons (d). Predicted interactions between the overlapping genes, with green, red and blue rings indicating the genes in the Venn diagram (e). The thickness of the connecting lines between the genes indicates the probability of the interaction. Genes coloured in blue and yellow in the predicted network indicate higher and lower expression in mature stage respectively.

To identify gene expression changes associated with the immature–mature transition, we compared immature individuals from both immature time points (*Immature1* and *2*) with those from the mature time point. We identified 50 DE genes between mature and immature individuals when both *vgll3* genotypes were combined, and when considering *vgll3* genotypes separately, 41 and 16 DE genes in vgll3*LL and vgll3*EE genotypes respectively (Figure 2a–c). Furthermore, we found a general tendency towards genes with increased expression in adipose tissue of mature males, which indicates higher transcriptional activation of the studied genes during maturation. This tendency was observed in the combined genotypes and vgll3*LL comparisons. In vgll3*EE individuals, 10 of the 16 DE genes between immature versus mature individuals had higher expression in the matures, whereas all the DE genes in vgll3*LL individuals had higher expression in the matures (Figure 2a–c). Across all three comparisons, expression patterns of three of the DE genes, *admc*, *col12a1a* and *pgra*, were independent of *vgll3* genotype, and all these genes showed increased expression in the mature males (Figure 2a–d). Importantly, *yap1*, the direct opposing interactor of *vgll3*, showed a similar pattern, that is, increased expression in mature vgll3*LL individuals (Figure 2b). We further investigated potential functional/molecular interactions between DE genes showing *vgll3* genotype‐specific differential expression (coloured numbers in Figure 2d). The predicted interactions between these genes revealed that among the three DE genes across all comparisons, only *pgra* had interactions with other genes in the interaction network (Figure 2e). From the *vgll3* genotype specific comparisons, 17 and 8 DE genes within vgll3*LL and vgll3*EE individuals, respectively, were found to be connected in the interaction network (circled in blue and green in Figure 2e). Moreover, *pgra* was connected to both the vgll3*LL and vgll3*EE interaction networks when genotypes were assessed separately through *snaia* and *foxo1c* respectively. Other genes with a high number of interactions in the network included *ets1a*, *lats2a/b* and *wwtr1a/b* in the vgll3*LL interaction network, and *mob1ab*, *tead1a* and *frmd6b* in the vgll3*EE network (Figure 2e).

**FIGURE 3:**
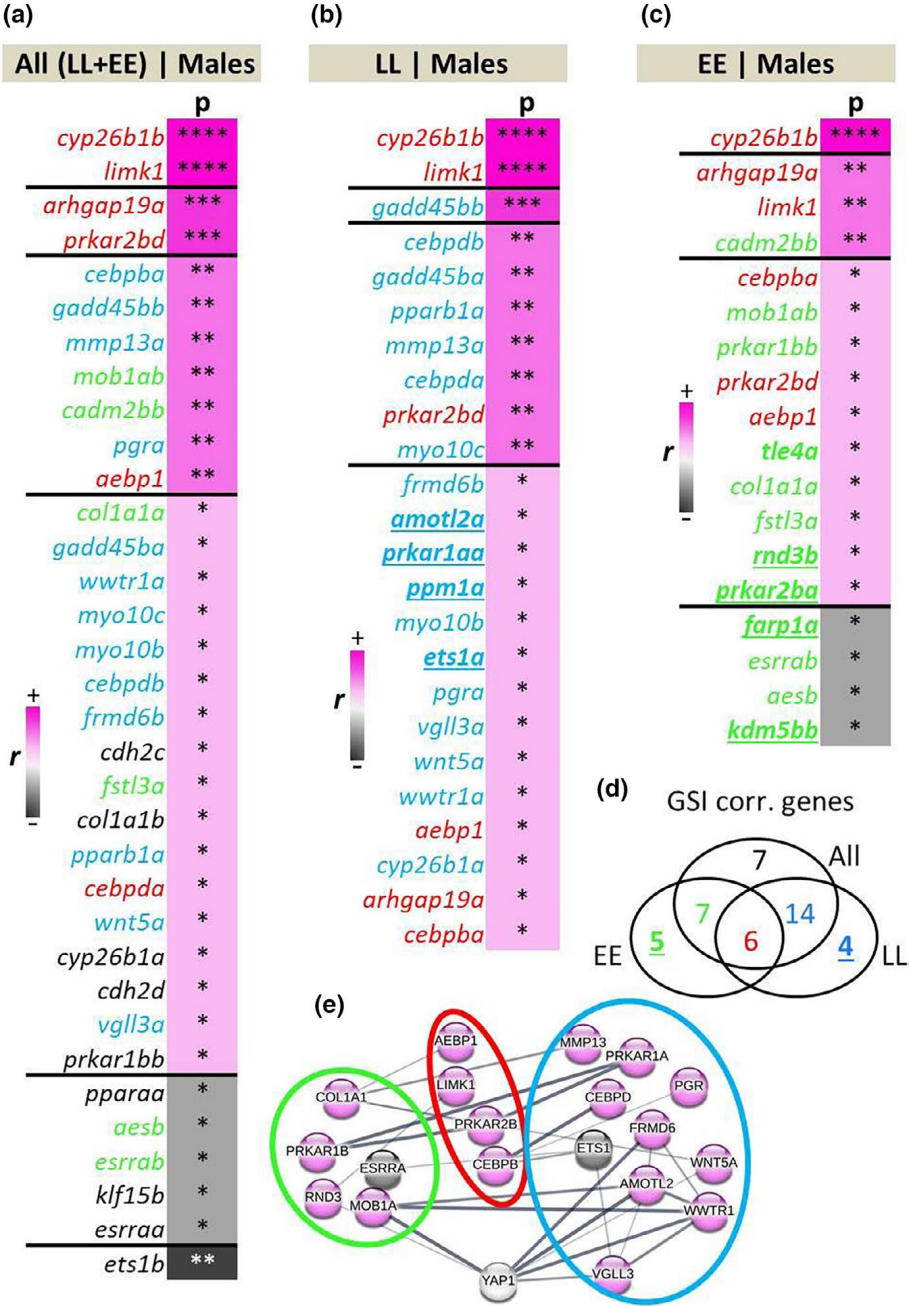
Genes showing GSI correlated expression and their predicted interactions in adipose tissue. Ranking of significant Pearson correlations between gene expression and GSI in salmon adipose tissue across both *vgll3* genotypes (a), within vgll3*LL (b) and vgll3*EE (c) genotype individuals. The Venn diagram depicts the numbers of significantly correlated genes unique or overlapping between the comparisons (d). Predicted interactions between the overlapping genes specified with green, red and blue rings indicating genes in the Venn diagram (e). EE and LL indicate vgll3*EE and vgll3*LL genotypes, respectively, and *p* and *r* imply *p*‐values (*<.05; **<.01; ***<.001; ****<.0001) and Pearson correlation coefficient. The thickness of the connecting lines between the genes indicates the probability of the interaction.

Next, we explored the gonadal development stage (using GSI) associated expression of the entire gene panel in order to identify genes with expression patterns in adipose tissue tightly linked with maturation stage. Interestingly, we found the expression of six genes; *aebp1*, *arhgap19a*, *cebpda*, *cyp26b1b*, *limk1* and *prkar2bd* to be positively correlated with GSI irrespective of *vgll3* genotype (Figure 3a–d). Furthermore, two of these genes, *cyp26b1b* and *limk1*, displayed the most significant positive expression correlations among all the genes within samples of each genotype. In general, most of the identified correlations were positive regardless of how *vgll3* genotypes were grouped (Figure 3a–c). Four genes among vgll3*EE genotype individuals showed negative expression correlations with increasing GSI. Among these, two genes, *esrrab* and *aesb*, were shared between vgll3*EE genotype and both genotypes together. Interestingly, among vgll3*LL individuals, expression of *vgll3a* was found to be among the genes positively correlated with GSI. The predicted interactions between the significantly correlated genes showed genotype‐specific expression correlations (coloured numbers in Figure 3d). These revealed potential interaction networks consisting of nine and 10 genes respectively for vgll3*LL and vgll3*EE genotype individuals and four overlapping genes across all groups (Figure 3e). The genes with the strongest and/or highest number of connections in the network include *amotl2a*, *frmd6b* and *wwtr1a* in the vgll3*LL interaction network, and *col1a1a* and *mob1ab* in the vgll3*EE network (Figure 3e).

### 3.3 Identification of gene coexpression networks

**FIGURE 4:**
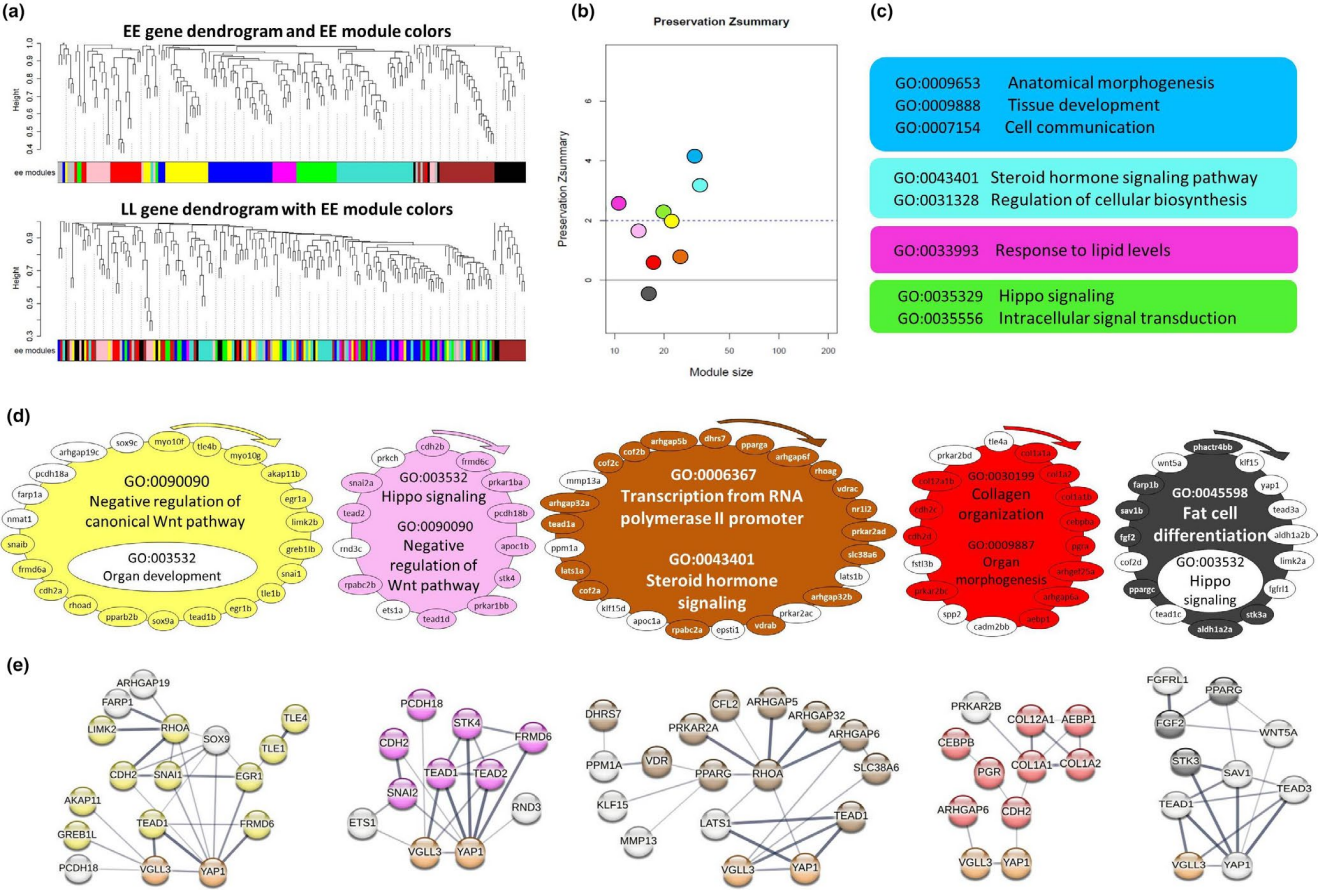
Coexpression analysis of vgll3*EE genotype in adipose tissue of male Atlantic salmon. (a) Visual representation of vgll3*EE module preservation in vgll3*LL individuals. The dendrograms represent average linkage clustering tree based on topological overlap distance in gene expression profiles. The lower panels of the dendrograms represent colours that correspond to the vgll3*EE clustered coexpression modules (GCMs). Top: vgll3*EE GCMs with assigned colours. Bottom: visual representation of the lack of preservation of vgll3*EE GCMs genes in vgll3*LL genotype individuals. (b) Preservation Zsummary scores in the vgll3*LL GCMs for vgll3*EE GCMs (colours represent vgll3*EE GCMs). Zsummary <2 represents lack of preservation (dotted blue line) and Zsummary between 2 and 10 implies moderate preservation. (c) The top hit GO biological processes among the most significantly enriched GOs in each of the well‐preserved coexpression modules. (d) The genes in each of the identified GCMs for vgll3*EE genotype with least preservation in vgll3*LL. The genes without colour in each module are those showing no preserved expression correlation in vgll3*LL individuals and the clockwise arrows above each GCM indicate the direction of genes with highest to lowest expression correlations with other genes within that GCM. For each of the GCMs, the top enriched GOs are represented, and GOs without colour were no longer enriched after removal of the genes without colours. (e) Predicted interactions between the genes within each of the less‐preserved GCMs. Different levels of thickness in the connecting lines between the genes indicates the probability of the interaction.

In order to gain better overview of transcriptional dynamics of Hippo pathway components and their known interacting genes, we applied network‐based co‐expression analyses in which genotype dependent changes in each network could be tracked. To do this, we first built GCN in the adipose tissue of each *vgll3* genotype and then investigated the preservation of the identified gene co‐expression modules or GCMs (within each GCN) between the genotypes. In other words, we defined the GCN in one genotype (vgll3*EE or vgll3*LL) and then assessed the preservation of its modules in the other genotype (vgll3*LL or vgll3*EE) respectively. We identified nine GCMs for vgll3*EE (Figure 4a,b) of which four, blue, turquoise, magenta and green, showed moderate preservation (Zsummary >2) in vgll3*LL, that is, most of the genes in each GCM have significant expression correlations in both genotypes. The other five GCMs (yellow, pink brown, red and black) showed a low level of preservation between *vgll3* genotypes (Zsummary <2) (Figure 4a).

GO Biological Process enrichment analysis was conducted for each of the GCMs separately in order to identify the key biological processes the genes in each GCM are involved in. Enriched GO terms for the moderately preserved GCMs included tissue development and morphogenesis, steroid hormone signalling and Hippo signalling pathways as well as response to lipid levels (Figure 4c). In the low preserved GCMs, we found coupling of Hippo pathway with fat cell differentiation in the black GCM and with negative regulation of canonical Wnt pathway in the pink GCM (Figure 4c). The black GCM showed the least preservation between the genotypes with most of its genes showing no coexpression preservation in vgll3*LL genotype individuals (genes lacking colour in each GCM in Figure 4d). Removal of unpreserved genes in the yellow and black GCNs led to loss of significance of one GO in each GCM; organ development in the yellow and Hippo signalling in the black GCM (non‐coloured GOs in Figure 4d). The removal of the unpreserved genes in the other GCNs did not change their enriched GOs (Figure 4d).

Knowledge‐based interactome prediction analysis using genes within each GCM was applied in order to identify potential interactions between the genes as well as hub genes with highest number of interactions. The prediction of interactions between the genes within each GCM revealed that except in the red GCM, all the other GCMs had at least one gene among those not showing coexpression preservation that had direct interaction with *vgll3/yap1* (represented with lines directly connecting the non‐coloured genes with *vgll3/yap1*; Figure 4e). This was more pronounced in the black GCM where four genes (*sav1b*, *tead1c*, *tead3a* and *wnt5a*) had direct interactions with *vgll3/yap1* (Figure 4e). Importantly, *yap1* itself lost coexpression preservation in the vgll3*LL GCM, indicating the involvement of yap1, the major inhibitor of the Hippo pathway, in differences between the *vgll3* genotypes in the lowest preserved GCM.

**FIGURE 5:**
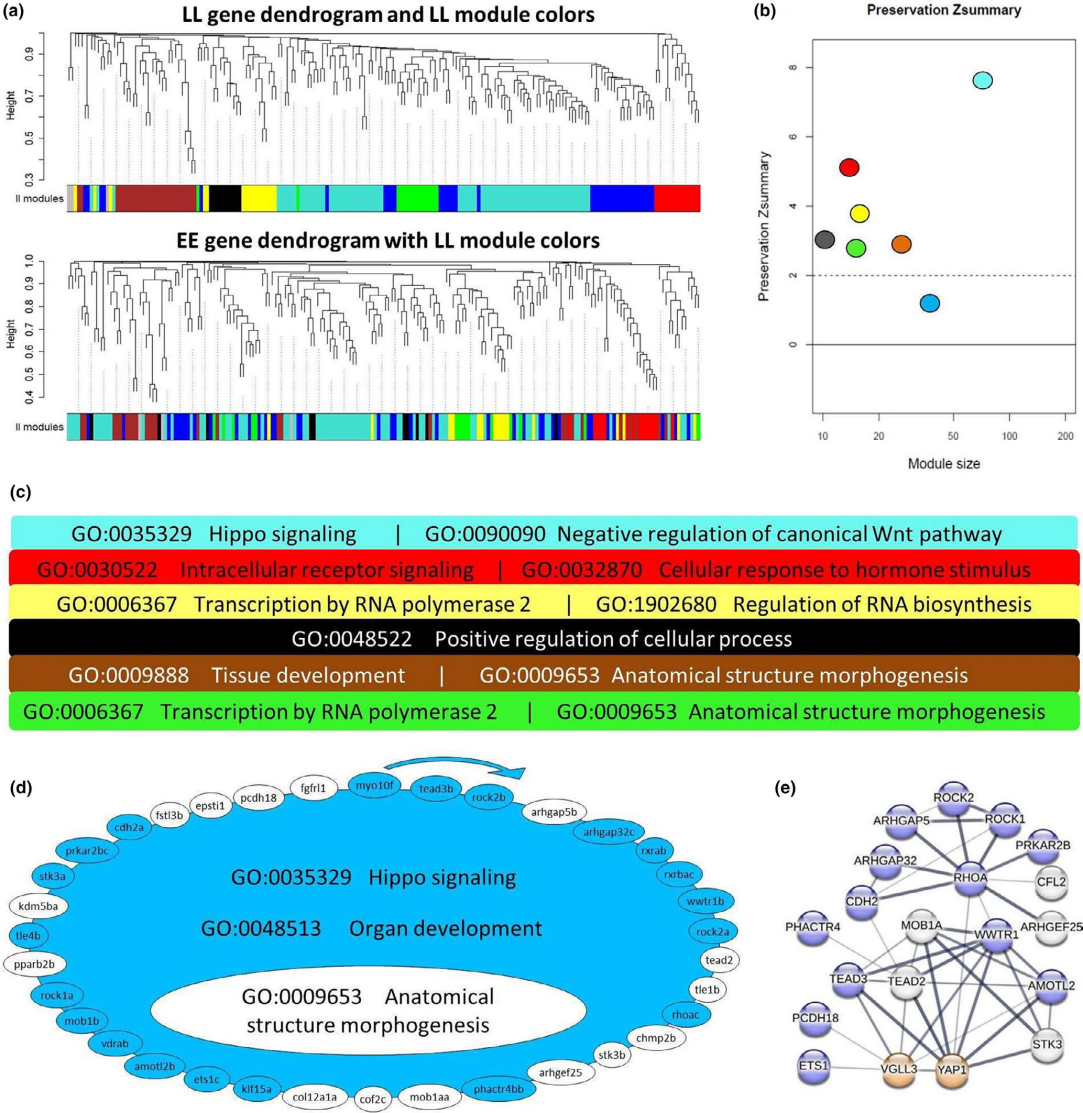
Coexpression analysis of vgll3*LL genotype in adipose tissue of male Atlantic salmon. (a) Visual representation of vgll3*LL module preservation in vgll3*EE individuals. The dendrograms represent average linkage clustering tree based on topological overlap distance in gene expression profile. The lower panels of the dendrograms represent colours that correspond to the clustered coexpression modules (GCMs). Top: vgll3*LL GCMs with assigned colours. Bottom: visual representation of preservation of vgll3*LL module genes in vgll3*EE genotype. (b) The colours represent identified vgll3*LL GCMs. Preservation of genes found in vgll3*LL GCMs in the vgll3*EE GCMs was calculated and Zsummary <2 represents lack of preservation (dotted blue line). Zsummary between 2 and 10 implies moderate preservation. (c) The most significant gene ontologies (GO) enriched in each of the well‐preserved coexpression modules. (d) The genes in each of the identified coexpression modules for vgll3*LL genotype with least preservation in vgll3*EE. The genes without colour in each module have lost their preserved expression correlation in vgll3*EE individuals and the clockwise arrows above each GCM indicate the direction of genes with highest to lowest expression correlations with other genes within that GCM. For each of the module, the most significant enriched GOs are represented, and GOs without colour have lost their significance after removal of the genes without colours. (e) Predicted interactions between the genes within each of the less‐preserved GCMs. The connecting lines between the genes with different levels of thickness indicate the probability of the interaction.

In vgll3*LL individuals, we found seven GCMs and among them only the blue GCM showed a low level of preservation (Zsummary <2) compared to vgll3*EE individuals (Figure 5a,b). The turquoise GCM was the largest with 74 co‐expressed genes and also with the highest preservation level between the genotypes (Zsummary >7). Interestingly, the turquoise GCM included genes encoding components of the Hippo signalling pathway and genes that negatively regulate the canonical Wnt pathway (Figure 5c), indicating potential interactions between Hippo and Wnt pathway in vgll3*LL individuals. We observed such connections between these pathways in a GCM of vgll3*EE as well (the pink GCM in Figure 4d), however, the number of genes in the pink GCM (14 genes) was much less than the turquoise GCM (76 genes) (Figures 4b and 5b). This may indicate more extensive connections between regulators of Wnt pathway and components of Hippo pathway in vgll3*LL individuals. On the other hand, the blue GCM, which was the least preserved GCM in vgll3*LL, appeared as the second largest with 37 genes (Figure 5b,d). This may indicate that the Hippo pathway is more affected and/or plays a more extensive role in the adipose tissue of vgll3*LL individuals. The blue GCM displayed coupling of genes within Hippo signalling pathway with organ development and morphogenesis (Figure 5d), but removal of unpreserved genes led to no significant enrichment of the GO linked to tissue morphogenesis (non‐coloured genes and GO in Figure 5d). The prediction of interactions between the genes within the blue GCM revealed that three genes that lost their coexpression preservation, *mob1aa*, *stk3b* and *tead2*, had direct interactions with *vgll3*/*yap1* (Figure 5e).

## 4 DISCUSSION

Understanding the molecular basis of life‐history traits and their interaction with the environment is crucial to predict organismal fitness in future climates. In this study, we aimed to uncover transcriptional effects of alternative maturation age genotypes of the *vgll3* gene, a major determinant of age at sexual maturity in Atlantic salmon (Barson et al., 2015) and also a main co‐factor of the Hippo pathway (Hori et al., 2020), in adipose tissue of this species. To do this, we investigated the expression differences of genes encoding adipogenesis related factors, Hippo pathway components and their associated interacting partners/signals between *early* (E) or *late* (L) maturing *vgll3* alleles using a custom‐made Nanostring platform. This allowed us to predict the regulatory dynamics of Hippo pathway signalling in the adipose tissue of Atlantic salmon that has a seasonal breeding strategy tightly associated with lipid metabolic status. Thus, the study of distinct *vgll3* genotypes provides understanding of the molecular links among lipid metabolism, Hippo pathway activity and sexual maturation – a life‐history trait of high ecological importance. Our findings not only imply that *vgll3* and its associated pathway (Hippo) have extensive effects on transcriptional changes in adipose tissue in relation to sexual maturation of Atlantic salmon, but also suggest *vgll3*'s role in linking adipogenesis and seasonal, likely maturation related, changes in this species.

### 4.1 Gene expression patterns at the immature stage reflect higher lipid storage capacity of vgll3*EE individuals in the spring

A recent study showed a link between *vgll3* genotypes and lipid storage in the liver of immature males of Atlantic salmon during seasonal changes (from spring to autumn) (House, Debes, Holopainen, et al., 2023). A possible hepatic scenario was proposed based on the differences observed between *vgll3* genotypes; that is, vgll3*EE individuals storing larger lipid droplets in earlier/warmer season (spring), whereas vgll3*LL individuals storing in a later/colder season (autumn) in their liver (House, Debes, Holopainen, et al., 2023). Our adipose tissue gene expression results support this proposed hepatic scenario in the immature stage. However, the *vgll3* genotypic associations were reversed in the adipose of mature stage individuals. Our results suggest that vgll3*LL individuals are unlikely to store larger lipid droplets during the spring due to increased expression of a RhoA encoding gene (*rhoad*) in their adipocytes. RhoA is a well‐known key suppressor of adipogenesis and lipid droplet storage in mammalian cells (Meyers et al., 2005), and has also been suggested to be inhibited by *vgll3* dependent activation of Hippo pathway (Kurko et al., 2020). Vgll3 itself has been reported as an inhibitor of adipocyte differentiation in mice (Halperin et al., 2013) and linked with body condition variation in Atlantic salmon (Debes et al., 2021). Thus, our finding here of higher expression of the adipogenesis suppressor, *rhoad*, in vgll3*LL individuals is consistent with the hepatic scenario suggested by House, Debes, Holopainen, et al. (2023) and House, Debes, Kurko, et al. (2023), indicating potentially similar regulatory pattern in the liver and adipose. Furthermore, we found vgll3*LL individuals to have higher expression of a paralogue gene (*rnd3b*) encoding RND3, a key lipolysis factor (Dankel et al., 2019). Lipolysis is a process in which lipid droplets are utilised (the opposite to lipogenesis) (Ducharme & Bickel, 2008), and thus this finding may also supports the hepatic scenario proposed by (House, Debes, Holopainen, et al., 2023; House, Debes, Kurko, et al., 2023), whereby immature vgll3*EE individuals store larger lipid droplets during the spring.

### 4.2 Gene expression patterns at the mature stage reflect higher lipid storage capacity of vgll3*LL individuals in the early autumn

In mature males, the higher expression of major adipogenic factors *snai2a*, *cebpab* and *ajubab*, as well as reduced expression of lipolysis factors; *rnd3a* and *pparaa* in vgll3*LL individuals indicates an opposite pattern in mature compared to immature males (i.e. lower lipid storage capacity of vgll3*LL individuals in immature individuals). The observed pattern in mature individuals suggests that vgll3*LL individuals store larger lipid droplets in autumn compared to vgll3*EE individuals, suggesting an opposite shift between the genotypes from immature to mature status. A key component of the interacting hub among the differentially expressed genes between the genotypes in the mature stage was a paralogue of *Snai2*/*Slug* gene (*snai2a*) (Figure 1e,f). Interestingly, *Snai2* is known as an inducer of adipocyte differentiation and lipid accumulation (Pérez‐Mancera et al., 2007). Similarly, a paralogue of C/EBPα encoding gene (*cebpab*) with predicted interaction with Snai2 displayed higher expression in vgll3*LL individuals (Figure 1f). C/EBPα is a major inducer of late stage adipocyte differentiation, and in mammalian cells, its expression is directly promoted by the transcriptional co‐regulator Ajuba (Yan et al., 2022). Consistently, a paralogue gene encoding Ajuba (*ajubab*) was also among the genes with higher expression in vgll3*LL individuals (Figure 1f). In mammals, *Ajuba* is a direct transcriptional target gene for Yap1 during cell proliferation and differentiation, and increased activity of Yap1 induces *Ajuba* expression (Lange et al., 2015). Moreover, in contrast to the immature stage, we found lower expression of *rnd3a* in vgll3*LL compared to vgll3*EE individuals at the mature time point (Figure 1f). RND3 is an inhibitor of Rho kinase (ROCK) signalling and an important regulator of lipid metabolism in adipocytes via induction of lipolysis (Dankel et al., 2019). It has been shown that RND3 exhibits inhibitory interaction with ROCK1, both through direct protein binding and transcriptional regulation (Dankel et al., 2019; Wu et al., 2021). Interestingly, genetic variations in Rock1 have been found to be associated with seasonal maturation timing in Chinook salmon and Steelhead (Collins et al., 2020; Narum et al., 2018). In agreement with the reduced expression of the lipolysis factor, *rnd3a*, we also observed reduced expression of *pparaa* in vgll3*LL individuals which encodes a major receptor activating lipolysis (PPARα) in adipose and liver (Guzmán et al., 2004). Combined, these expression patterns suggest higher lipid storage capacity of vgll3*LL individuals in the early autumn at mature stage.

### 4.3 Gene expression patterns reflect reduced lipid storage capacity at the mature stage in both *vgll3* genotypes but with less Hippo pathway dependency in vgll3*EE

Comparison of expression patterns between immature and mature stages suggest reduced adipogenesis and increased lipolysis in the mature males, and subsequently, the utilisation of lipid droplets to invest energy in sexual maturation. Comparing all immature and mature individuals, only one gene encoding progesterone receptor (*pgra*) and a paralogue gene of its downstream target, adrenomedullin (*admc*) were differentially expressed independently of *vgll3* genotype across all the immature versus mature comparisons. In zebrafish, differential expression of *pgra* in association with activity of leptin signal, an adipogenesis regulating pathway, has been indicated in sexual maturation (Tsakoumis et al., 2022). In mammals, it has been recently demonstrated that PGR can act as inhibitor of adipogenesis (Liu et al., 2021) and also be a stimulator of lipolysis (Zhang et al., 2021). We observed similar patterns between mature and immature individuals within each *vgll3* genotype based on the expression pattern of genes involved in lipogenesis and lipolysis. However, it seems that the reduced adipogenesis (and lipogenesis) of mature individuals is the result of different mechanisms within each genotype. In vgll3*LL individuals, the vast majority of DE genes exhibited an increase in expression at the mature stage (including *lats2* and *yap1*). This may suggest that lipolysis in mature vgll3*LL individuals might be mediated through increased expression of a major Hippo pathway kinase, *lats2*, which is also known to promote lipolysis (El‐Merahbi et al., 2020). In vgll3*EE mature individuals, other regulators independent of the Hippo pathway such as FOXO1 and ESRRα (encoded by *foxo1c* and *esrrab*) appeared to be the mediators of the potential processes of increased lipolysis and reduced lipogenesis. FOXO1 has a critical role in transcriptional induction of the major adipocyte lipolysis enzyme (adipose triglyceride lipase ATGL) (Chakrabarti & Kandror, 2009). Oestrogen‐related receptor α (ESRRα) is a nuclear receptor that promotes adipocyte differentiation by regulating the expression of various adipogenesis‐related genes (Ijichi et al., 2007). Hence, the higher expression of *foxo1c* and the lower expression of *esrrab* in vgll3*EE mature compared to immature individuals could be another indicator of reduced adipogenesis and increased lipolysis in the mature males. However, a gene encoding C/EBP‐β (*cebpba*), an early stage inducer of pre‐adipocyte proliferation was also upregulated in the mature vgll3*EE individuals (Merrett et al., 2020). Taken together, this suggests that the adipose tissue of the mature males in this genotype might have higher number of undifferentiated pre‐adipocytes, however, histological studies are required to approve this hypothesis. The perpetuation of undifferentiated stage in pre‐adipocytes might be also linked to the inhibitory role of *vgll3* during adipogenesis, which mainly blocks the terminal stage of adipocyte differentiation (Halperin et al., 2013).

### 4.4 Gene expression‐GSI correlations suggests reduced lipid storage capacity at the mature stage in both *vgll3* genotypes

**FIGURE 6:**
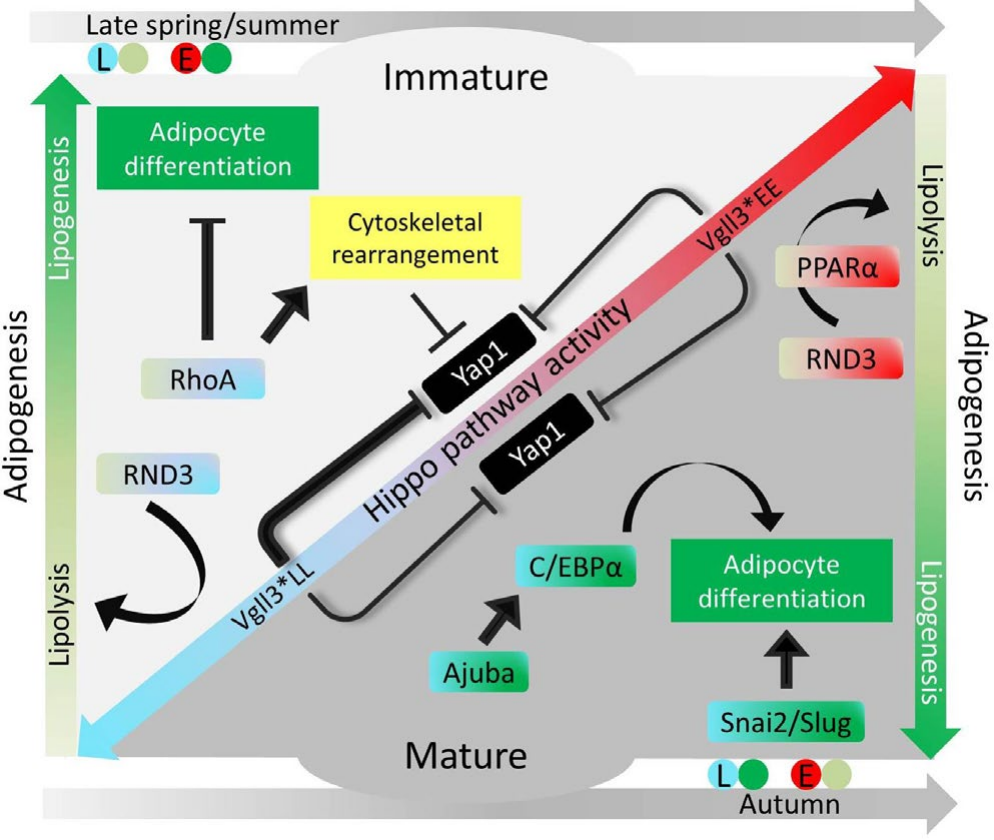
A schematic representation of the main findings linking vgll3 genotype and Hippo pathway components to different processes in the adipose tissue of Atlantic salmon. Colours indicate Hippo pathway modes of activity through the late and early maturation *vgll3* genotypes (vgll3*LL: light blue and vgll3*EE: red), as well as distinct adipogenic/lipogenic (dark green) and lipolytic (light green) processes. Genes are colour coded according to *vgll3* genotype (blue or red) and lipogenesis/lysis statuses (dark or light green) indicating higher expression in the respective genotype and/or the resulting lipogenesis/lysis outcome of their higher expression. Arrow heads indicate activation whereas blocked head implies inhibition.

To gain more insight into the molecular details of the effects of maturation on adipose gene expression, we also conducted correlation analysis between gene expression and gonadal maturation level (GSI score). A notable gene among those with the strongest positive correlations with gonadal maturation level across genotypes was a gene encoding LIM domain kinase 1 (*limk1*), which is an inhibitor of adipocyte differentiation through modulation of intracellular structural organisation required during the differentiation (Chen et al., 2018). Interestingly, *limk1* is a direct downstream target of PGR in humans (Mazur et al., 2015), thus this result is consistent with the potential role of PGR in mature individuals generally showing lower lipidogenesis and higher lipolysis capacity (see above). This suggests reduced adipogenesis in the mature males of both genotypes. However, clear expression pattern differences between the *vgll3* genotypes were observed as well. For instance, in mature vgll3*LL individuals, we found a positive correlation between *vgll3a* expression and gonadal maturation suggesting a more direct role of *vgll3a* itself in inhibition of adipogenesis during maturation in this genotype (Halperin et al., 2013). In vgll3*EE individuals, we observed negative correlation between *esrrab* expression and gonadal maturation suggesting diminished ESRRα‐mediated adipocyte differentiation, as ESRRα is a known inducer of adipocyte differentiation (Ijichi et al., 2007). Hence, this may indicate differential expression of ESRRα to be an important mechanism for reduced adipogenesis in vgll3*EE individuals (Figure 2), and also suggests that the reduced adipogenesis in this genotype is less dependent on the Hippo pathway. A summary of all abovementioned findings in respect to salmon maturation, seasonal effects, and adipogenesis is depicted in Figure 6.

### 4.5 Gene expression network analysis suggests differing Hippo pathway associations in vgll3*EE and vgll3*LL individuals

Hippo pathway is emerging as a key molecular signal in balancing adipocyte proliferation versus differentiation, and the activity of Yap has been found to be indispensable during adipogenesis (Ardestani et al., 2018). We conducted GCN analysis to identify genotype‐specific differences within the network of co‐expressed genes in adipose tissue, and found that in vgll3*LL individuals, there was a lack of association between expression of the Hippo pathway components and genes involved in adipocyte differentiation. In the GCM identified in vgll3*EE, with the lowest preservation level between the genotypes (the black GCM in Figure 5), we observed a lack of a link between the GO terms related to fat cell differentiation from the GO of the Hippo pathway. Interestingly, several genes encoding major components of Hippo pathway were among the genes that lacked coexpression preservation in vgll3*LL (*tead1c*, *tead3a*, *sav1b* and *yap1*). Sav1, an inhibitor of Yap, has been shown to play a main role in enhancing adipocyte differentiation in mammals (Ardestani et al., 2018). Thus, these findings in the black GCM suggest that there are distinct transcriptional patterns of Hippo pathway components between the *vgll3* genotypes in relation to adipogenesis, which might be a result of different *sav1b* and *yap1* regulatory functions in these genotypes. However, a direct regulatory connection between Sav1 and Vgll3 has not been investigated in any species. In the yellow GCM, we found no association between the GO term related to negative regulation of Wnt signalling and the GO of organ development. Strikingly, among the genes in this GCM showing lack of expression preservation between the genotypes was a negative regulator of Wnt signalling, *cdh2* (encoding N‐cadherin), which is known to enhance adipogenesis by inhibiting Wnt signal (Haÿ et al., 2014). Moreover, *cdh2* is a well‐known regulator of organ development (García‐Castro et al., 2000), and also a downstream transcriptional target of vgll3 (together with *pcdh18a* which also lacks coexpression preservation in the yellow GCM) (Simon et al., 2017). Finally, *cdh2* appeared to be critical for structural/mechanical regulation of Hippo pathway during adipose tissue development (Nardone et al., 2017).

In vgll3*EE individuals, we found a lack of association between expression of the Hippo pathway components and genes involved in structural morphogenesis. Among the identified GCMs in vgll3*LL genotype individuals, only one GCM containing genes involved in Hippo pathway, organ development and structural morphogenesis (depicted in blue in Figure 5) showed a low level of preservation between the genotypes. The adipose tissue morphogenesis and structural remodelling are major contributors to metabolic, functional and adaptive changes in this tissue (Choe et al., 2016; Liu et al., 2020). When the blue GCM was compared to its structure in vgll3*EE genotype individuals, there was a lack of co‐expression preservation for genes involved in tissue morphogenesis and remodelling such as *cof2c* (*CFL2*), *col12a1a* and *fstl3b* (Ahi, 2016; Chen et al., 2023; Galkin et al., 2011; Li et al., 2023) and adipogenesis such as *fgfrl1* (Lecaudey et al., 2021; Wang et al., 2020). The preservation loss in this GCM resulted in the dissociation of the GO term related to structural morphogenesis from the other GOs (Hippo pathway and organ development) (Figure 5). In addition, there was also an absence of coexpression preservation in vgll3*EE individuals for several members of Hippo pathway (*mob1aa*, *tead2a* and *stk3b*). Since the pathway itself is critical for adipose tissue remodelling and morphological modification (Lecoutre et al., 2022; Shen et al., 2022), the lack of correlations with genes involved in morphogenesis is most likely a downstream consequence of transcriptional differences between the genotypes in these three key components of the Hippo pathway. Reciprocally, morphological/structural changes at the intra‐ and extra cellular levels can mediate their effects on adipose tissue by regulating Hippo pathway signalling (Liu et al., 2022; Seo & Kim, 2018). These findings suggest that the differing role of the Hippo pathway between *vgll3* genotypes potentially results in distinct structural/morphological changes in the adipose tissue of the two *vgll3* genotypes in salmon, and these differences are likely to be dependent on Yap/Taz transcriptional activity. Another plausible scenario could be that the different *vgll3* genotypes exhibit variations in the timing (heterochrony) of their interactions with the components of the Hippo pathway. Future studies incorporating additional time‐points for each season could be crucial in investigating this possibility.

## 5 CONCLUSIONS

This study provides important molecular evidence linking ecological change (seasonality), physiological process (lipid metabolism/storage) and genotype‐linked life‐history strategy variation (sexual maturation). Our findings provide gene expression evidence supporting the previously proposed scenario in which Atlantic salmon immature males exhibiting genotype specific lipid profiles with vgll3*EE individuals increasing lipid storage between spring and autumn while vgll3*LL individuals do the opposite. Furthermore, such a pattern seems to be reversed (i.e. vgll3*EE individuals decreasing lipid storage) in the mature individuals during autumn. At the molecular level, our results suggest that certain components of the Hippo signalling pathway (e.g. *yap1*, *sav1*, *lats2*, etc.) are potentially major drivers of the differing effects of *vgll3* genotypes on the adipose tissue gene expression. However, more detailed functional assessments are required to better validate these suggested differences. Additional useful follow‐up investigations include assessment of expression patterns in vgll3 heterozygotes, as well as whether similar genotype‐specific expression patters are observed in females.

## AUTHOR CONTRIBUTIONS

EPA, CRP, JPV, JK and PVD conceived the study; JPV, PVD and CRP reared and sampled the fish; CRP and JE provided resources; AR, EPA and JPV performed experiments; EPA, JPV, PS and JK developed methodology and analysed the data; EPA, JPV and CRP interpreted results of the experiments; EPA, JPV and CRP drafted the manuscript, with EPA having the main contribution, and all authors approved the final version of manuscript.

## FUNDING INFORMATION

Funding was provided by Academy of Finland (grant numbers 307593, 302873, 327255 and 342851), and the European Research Council under the European Articles Union's Horizon 2020 and Horizon Europe research and innovation programs (grant numbers 742312 and 101054307). Views and opinions expressed are however those of the authors only and do not necessarily reflect those of the European Union or the European Research Council Executive Agency. Neither the European Union nor the granting authority can be held responsible for them.

## CONFLICT OF INTEREST STATEMENT

Authors declare no competing interests.

## ETHICS STATEMENT

Animal experimentation followed European Union Directive 2010/63/EU under licence ESAVI/35841/2020 granted by the Animal Experiment Board in Finland (ELLA).

## Supporting information


File S1.

File S2.

## Data Availability

All the gene expression data generated during this study are included in this article as File S1 and S2.
